# Missing data and sensitivity analysis for binary data with implications for sample size and power of randomized clinical trials

**DOI:** 10.1002/sim.8428

**Published:** 2019-11-14

**Authors:** Thomas Cook, Ryan Zea

**Affiliations:** ^1^ Department of Biostatistics and Medical Informatics University of Wisconsin‐Madison Madison Wisconsin

**Keywords:** missing data, power, randomized controlled trial, sample size, sensitivity analysis

## Abstract

Despite our best efforts, missing outcomes are common in randomized controlled clinical trials. The National Research Council's Committee on National Statistics panel report titled *The Prevention and Treatment of Missing Data in Clinical Trials* noted that further research is required to assess the impact of missing data on the power of clinical trials and how to set useful target rates and acceptable rates of missing data in clinical trials. In this article, using binary responses for illustration, we establish that conclusions based on statistical analyses that include only complete cases can be seriously misleading, and that the adverse impact of missing data grows not only with increasing rates of missingness but also with increasing sample size. We illustrate how principled sensitivity analysis can be used to assess the robustness of the conclusions. Finally, we illustrate how sample sizes can be adjusted to account for expected rates of missingness. We find that when sensitivity analyses are considered as part of the primary analysis, the required adjustments to the sample size are dramatically larger than those that are traditionally used. Furthermore, in some cases, especially in large trials with small target effect sizes, it is impossible to achieve the desired power.

## 1 INTRODUCTION

Despite our best efforts, missing outcomes are common in randomized controlled clinical trials (RCTs). Numerous authors have commented on specific problems resulting from missing responses.^1^, ^2^ In 2010, the National Research Council's Committee on National Statistics issued a panel report titled *The Prevention and Treatment of Missing Data in Clinical Trials.* 3, 4 Among other things, this report stressed the importance of sensitivity analyses as an essential tool to assess the reliability of primary analyses. The report also lists a number of areas in need of further research, two of which are the following: the effect of missing data on the power of clinical trials and how to set useful target rates and acceptable rates of missing data in clinical trials. O'Neill and Temple^5^ also recommend new research into, among other things, “sample size calculations in the presence of missing data.”

In this article, we describe a causal framework that clarifies the underlying problem and attempt to provide partial answers to these questions within this framework. Briefly, in Section 2, we formulate the primary question using the Rubin causal model^6^, ^7^ and provide a justification for randomization as the basis for valid inference regarding the causal effect of treatment on the response. In Section 3, using the binary case as an example, we formulate a missingness model useful for both illustrating the underlying problem and conducting sensitivity analyses. In Section 4, we show that the complete case analysis easily fails, even when a small proportion of responses are missing. In Section 5, we illustrate the use of principled sensitivity analysis for a specific 2×2 table, and in Section 6, we illustrate the impact of sample size on the conclusions from a sensitivity analysis. In Section 7, we show how, when the primary analysis involves principled sensitivity analysis, the adjustment to the sample size required to maintain the desired power can be quite large, and in some cases, maintaining power is impossible. Finally, Section 8 summarizes our findings.

## 2 CAUSAL FRAMEWORK

For the purpose of this article, we will consider the primary efficacy goal of a randomized controlled trial to be to establish whether there is a *causal association* between assigned treatment and the response of interest. We do this using a frequentist hypothesis testing approach, although other approaches should lead to similar conclusions. We begin with the framework of the *Rubin Causal Model* 6 as described by Holland^7^.

Let u∈U represent an arbitrary subject from the population of interest, U, and *Y*_*t*_(*u*) and *Y*_*c*_(*u*) denote the potential responses were subject *u* to be assigned either treatment *t*, the experimental treatment, or treatment *c*, the control. That is, *Y*_*t*_(*u*) is the response that would be observed were subject *u* to be assigned treatment *t*, and *Y*_*c*_(*u*) the response that would be observed were subject *u* to be assigned treatment *c*. For simplicity, we define the *causal effect* of *t* relative to *c* for subject *u* to be the difference

$$Y_{t}(u)-Y_{c}(u).$$

The *population average causal effect* is the average over the population

(1) $$E[Y_{t}(u)-Y_{c}(u)]=E[Y_{t}(u)]-E[Y_{c}(u)],$$

where the expectation is over the population U, 1Note that other summary measures can also be used—odds ratios, risk ratios, etc.—but the discussion is more complicated, and our overall findings will be unchanged.and *E*[*Y*_*τ*_(*u*)], *τ*=*c*,*t*, is the population average had all subjects in the population been assigned *τ*. When *Y*_*τ*_(*u*)∈{0,1} is a binary response, *E*[*Y*_*τ*_(*u*)]=*p*_*τ*_, the population average probability that a subject fails if assigned treatment *τ*.

We wish to exploit the right‐hand side of (1) to estimate the population average causal effect from two different sets of subjects. Let *T*(*u*) be the treatment assigned to subject *u*. We require that we know 2It is impossible to determine from the data whether (2) holds. We require additional external information, eg, that treatment assignment is randomized, to determine this.that

(2) $$E[Y_{T(u)}(u)|T(u)=\tau ]=E[Y_{\tau }(u)],$$

which holds if *T*(*u*) is independent of {*Y*_*t*_(*u*),*Y*_*c*_(*u*)}. This is in turn guaranteed if *T*(*u*) is randomly assigned and, therefore, the mean of *Y*(*u*) over subjects *assigned* treatment *τ* is an unbiased estimate of *p*_*τ*_. Hence, if we have no missing responses, we can conduct valid inference regarding the average causal effect of *t* relative to *c*. We make two comments regarding Equation (2).

1. Satisfying Equation (2) is the sole reason for using randomization. Assuming that we have complete ascertainment of all responses, no other conditions are necessary to ensure valid causal conclusions. If treatments are *not* randomly assigned, it is unclear that Equation (2) holds.
2. Equation (2) does not imply that treatment groups need to be balanced with respect to important baseline factors. In fact, it is clear from this equation that approximate balance is merely a side effect of randomization and not necessary for valid causal inference.

Now suppose responses are missing for some subjects. Let *δ*_*τ*_(*u*)=*I*{*Y*_*τ*_(*u*) be observed,*T*(*u*)=*τ*} where *I*{·} is the indicator function. Unless data are missing at random (MAR),^8^ 3For the binary case that we consider in this article, this is strictly true. For nonbinary responses, it is possible for equality to hold even if MAR fails.

$$E[Y_{T(u)}(u)|T(u)=\tau ,\delta_{\tau }(u)=1]\neq E[Y_{\tau }(u)]$$

and therefore the mean of *Y*(*u*) over subjects assigned *τ* with nonmissing data is *not* an unbiased estimate of *E*[*Y*_*τ*_(*u*)] and inference based solely on nonmissing responses does not have a direct causal interpretation. This is the fundamental problem with missing data in randomized trials.

In the binary case, let *p*_*τ*_=*E*[*Y*_*τ*_(*u*)] and ${\tilde{p}}_{\tau }=E[Y_{T(u)}(u)|T(u)=\tau ,\delta_{\tau }(u)=1]$ and note that we have two distinct (statistical) null hypotheses

$$H_{0}:p_{t}=p_{c}$$

and

$${\tilde{H}}_{0}:{\tilde{p}}_{t}={\tilde{p}}_{c}.$$

Importantly, from a purely statistical point of view, these are equally valid hypotheses. The *only* distinction between them is that the first has a direct *scientific* interpretation while the second does not. By ignoring all observations with missing responses, “conventional” statistical analysis allows (correct!) inference regarding ${\tilde{H}}_{0}$, whereas missingness precludes “conventional” statistical inference regarding *H*_0_. Furthermore, were *H*_0_ to be true but ${\tilde{H}}_{0}$ false, a complete case analysis that rejects ${\tilde{H}}_{0}$ would be a correct rejection and hence *not* a type I error.

## 3 MISSINGNESS MODEL

In the remainder of this article, we will assume that *Y*_*τ*_(*u*)∈{0,1}, where *Y*_*τ*_(*u*)=1 represents failure and that treatments are randomly assigned. We reduce the observed data to that shown in Table 1 where *y*_*c*_ and *y*_*t*_ are the numbers of failures, *m*_*c*_ and *m*_*t*_ are the numbers of nonmissing responses, and *n*_*c*_ and *n*_*t*_ are the numbers randomized for treatments *c* and *t* respectively.

**Table 1:** Summary data for a binary response with missing responses

	***Y***_***τ***_ ** = 1,** ***δ***_***τ***_ ** = 1**	***δ***_***τ***_ ** = 1**	Total
*τ*=*c*	*y*_*c*_	*m*_*c*_	*n*_*c*_
*τ*=*t*	*y*_*t*_	*m*_*t*_	*n*_*t*_

Using a selection model,^9^ we denote the conditional probability that an observation is missing given *Y*_*τ*_(*u*)=0 by

$$\pi_{\tau }=E[1-\delta_{\tau }(u)|Y_{\tau }(u)=0]$$

and define *r*_*τ*_>0 by

$$r_{\tau }\pi_{\tau }=E[1-\delta_{\tau }(u)|Y_{\tau }(u)=1].$$

Note that *r*_*τ*_ is the ratio of the probability of missingness given *Y*_*τ*_(*u*)=1 to the probability of missingness given *Y*_*τ*_(*u*)=0. We denote the marginal missingness probabilities by *q*_*τ*_=*E*[1−*δ*_*τ*_(*u*)]=(1−*p*_*τ*_+*r*_*τ*_ *p*_*τ*_)*π*_*τ*_. We have that

$$E[y_{\tau }]=p_{\tau }(1-r_{\tau }\pi_{\tau })n_{\tau }$$

and

$$E[m_{\tau }]=(1-q_{\tau })n_{\tau }.$$

The failure probability among subjects with nonmissing responses in group *τ*, ${\tilde{p}}_{\tau }$ is

$${\tilde{p}}_{\tau }=E[Y_{\tau }(u)|\delta_{\tau }(u)=1]=\frac{p_{\tau }(1-r_{\tau }\pi_{\tau })}{1-(1-p_{\tau }+r_{\tau }p_{\tau })\pi_{\tau }}.$$

It is easily shown that the impact of deviations from MAR is completely characterized by the pair (*r*_*c*_,*r*_*t*_). When *r*_*c*_=*r*_*t*_=1, ${\tilde{p}}_{\tau }=p_{\tau }$ and missingness is MAR and *ignorable*.^10^ Values of *r*_*t*_ and *r*_*c*_ that are farther from 1 represent greater deviations from MAR.

Before proceeding, we note two things when the hypothesis *H*_0_ is true.

1. If observations are deliberately omitted from the analysis for subjects who are nonadherent to their assigned treatment thereby inducing missingness, except under the strong untestable (and likely implausible) assumption that subjects are *nonadherent at random*, the resulting analysis does not assess the causal effect of treatment. Specifically, a so‐called *per‐protocol* analysis *cannot* correct for nonadherence to assigned treatment.
2. As we show numerically in Section 4, if *H*_0_ is true (assigned treatment has no causal effect on failure), but ${\tilde{H}}_{0}$ is false, as the sample size increases, power for rejecting ${\tilde{H}}_{0}$ increases. Specifically, the adverse impact of missing data on statistical inference *increases* with sample size. Consequently, in large trials, even low rates of missingness can have a dramatic adverse effect on the credibility of the conclusions.

In general, the missing data mechanism will be unknown and while we cannot assume ignorability, we may be able to determine a range of *plausible* values of (*r*_*c*_,*r*_*t*_). Denote the region containing all plausible values by R. In the examples that follow, for purposes of illustration, we assume such regions exist and that they are elliptical on the $\gamma_{\tau }=\log r_{\tau }$ scale, with major axis along *γ*_*t*_=*γ*_*c*_, minor axis along *γ*_*t*_=−*γ*_*c*_, and eccentricity denoted by *e*. This form is selected assuming that *γ*_*c*_ and *γ*_*t*_ are more likely to have the same sign than opposite signs—if they have opposite signs, they are not far from zero—and that values of *γ*_*τ*_ with opposite signs are equally plausible. Other forms for R can certainly be used, but the general principles that we highlight in this article will hold for any form of R. One might also adopt approaches with a more Bayesian flavor by imposing prior probabilities to points (*r*_*c*_,*r*_*t*_). Again, however, regardless of the specific implementation, the overall conclusions that we draw in this article should apply, only the implementation will change.

**Figure 1:**
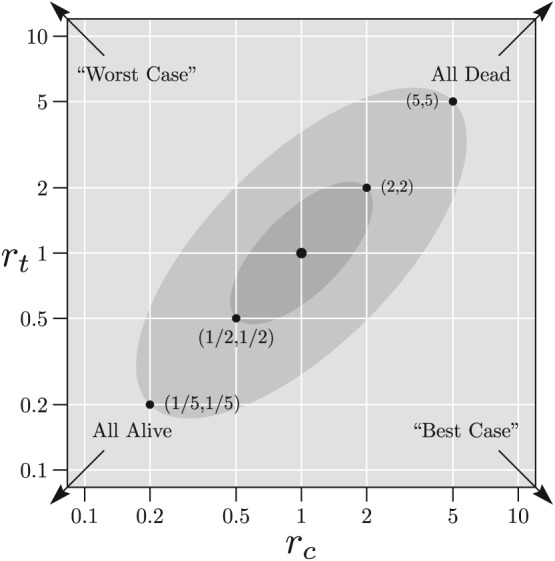
Example plausible regions shown on $\log r_{c}$ and $\log r_{t}$ scales where the outcomes are “dead” (failure) and “alive” (success). The lightly shaded ellipse shows the “skeptical region” whose boundary lies between the points (1/5, 1/5) and (5, 5). The darker shaded ellipse shows the “optimistic region” whose boundary lies between the points (1/2, 1/2) and (2, 2). The point (1, 1) corresponds to missing at random

Specifically, for fixed *e*∈[0,1), we consider elliptical regions of the form

$$R_{a}=\left\{(r_{c},r_{t}):{\left(\gamma_{t}+\gamma_{c}\right)}^{2}+\frac{{\left(\gamma_{t}-\gamma_{c}\right)}^{2}}{\left(1-e^{2}\right)}\leq 4{\left(\log a\right)}^{2}\right\}$$

for *a*>0. For example, if *γ*_*t*_=*γ*_*c*_, we have $|\gamma_{\tau }|\leq \log a$, and if *γ*_*t*_=−*γ*_*c*_, then $|\gamma_{\tau }|\leq \sqrt{1-e^{2}}\log a$. For illustration, we will select two specific values of *a* and fix *e*=0.9. For *a*=5, $R_{5}$ is the *skeptical* plausibility region, allowing large deviations from ignorability, and for *a*=2, $R_{2}$ with is the *optimistic* region allowing only small deviations from ignorability. These regions are shown in Figure 1. The point (1, 1) corresponds to MAR, the inner ellipse is $R_{2}$ and the outer ellipse is $R_{5}$. By “worst case,” we mean that all subjects in group *t* with missing responses are failures, whereas all subjects in group *c* with missing responses are successes. This is equivalent to letting *r*_*c*_→0 and *r*_*t*_→*∞*. Conversely, the opposite is true for the “best case” for which *r*_*c*_→*∞* and *r*_*t*_→0. “All Alive” means that all missing observations in both groups are successes, whereas “all dead” means that all missing observations in both groups are failures.

## 4 COMPLETE CASE ANALYSIS

The *Z*‐statistic corresponding to the (one‐sided) Pearson chi‐square test applied to the complete cases can be written as

(3) $$\tilde{Z}=\frac{{\hat{p}}_{c}-{\hat{p}}_{t}}{\sqrt{\bar{p}(1-\bar{p})(1/m_{t}+1/m_{c})}},$$

where ${\hat{p}}_{\tau }=y_{\tau }/m_{\tau }$ and $\bar{p}=(y_{c}+y_{t})/(m_{c}+m_{t})$.

Consider a complete case analysis of Table 1 using the *Z*‐statistic in (3) with rejection region $|\tilde{Z}|\geq 1.96$. As noted, this test has (asymptotic) type I error rate of 5% for ${\tilde{H}}_{0}$, however, if *H*_0_ is true but ${\tilde{H}}_{0}$ is false, the probability of rejection will exceed 5%. That is, it properly controls type I error rates for a scientifically uninteresting hypothesis, but is not an unbiased test of the hypothesis of interest, *H*_0_.

**Figure 2:**
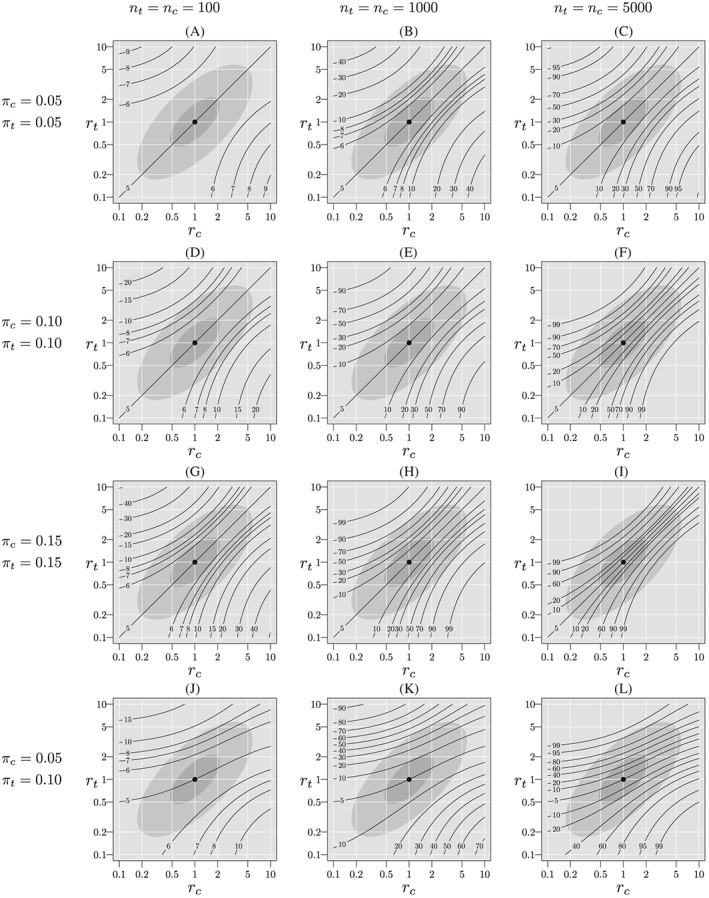
Probability of rejection of ${\tilde{H}}_{0}$ (%) when *H*_0_ is true, *p*_*t*_=*p*_*c*_=0.3, varying *π*_*t*_, *π*_*c*_, *n*_*t*_, and *n*_*c*_. The light gray ellipse corresponds to the skeptical plausibility region, $R_{5}$, and the darker gray ellipse corresponds to the optimistic region, $R_{2}$

Figure 2 shows contour lines for rejection probabilities (as percents) as a function of *r*_*c*_ and *r*_*t*_ when *p*_*t*_=*p*_*c*_=0.3, *π*_*c*_ and *π*_*t*_ are 0.05, 0.10, or 0.15, and *n*_*c*_=*n*_*t*_=100,1000,5000. For panel A, with sample size 100 per group and missingness rates of 5% per group, the rejection probabilities are only minimally above 5%; at most about 5.5% within $R_{5}$ and at most about 5.1% in $R_{2}$. As the sample size increases, however, the rejection probability increases. In panel C, with sample size 5000 per group, the rejection probability can exceed 30% in $R_{5}$ and 10% in $R_{2}$. Furthermore, as missingness rates increase, so do rejection probabilities. In panel I, with missingness rates of 15% and sample sizes of 5000 per group, rejection probabilities exceed 99% and 60% for regions $R_{5}$ and $R_{2}$, respectively.

Note further that when *π*_*t*_=*π*_*c*_ (rows 1, 2, and 3), and *r*_*t*_=*r*_*c*_ (the diagonal), the missingness mechanism does not depend on treatment, hence ${\tilde{H}}_{0}$ is true and the rejection probabilities equal the size of the test. Nonetheless, missingness rates that are equal should not lead us to conclude that the complete‐case analysis is appropriate. On the other hand, when *π*_*t*_≠*π*_*c*_ (panels J, K, and L), the missingness mechanism does depend on treatment unless r_*t*_=r_*c*_= 1, and for *r*_*t*_=*r*_*c*_≠1, the rejection probability exceeds the nominal size of the test. In all cases a similar pattern emerges, the rejection probabilities increase as sample size increases.

These results suggest that the rejection probabilities could be controlled by recalibrating the complete data critical value. However, this approach requires that we know the underlying values of *π*_*t*_ and *π*_*c*_. A more principled alternative, requiring only specification of R, is the formal *sensitivity analysis* discussed in the next section.

## 5 SENSITIVITY ANALYSIS FOR A 2×2 TABLE

For simplicity, for the remainder of this article, we will consider only one‐sided tests of *H*_0_. A formal sensitivity analysis uses the likelihood for the full Table 1, including the missing responses.

We formulate the likelihood using the logistic model

$$\log\frac{p_{\tau }}{1-p_{\tau }}=\zeta +\eta z_{\tau },$$

where *z*_*c*_=0 and *z*_*t*_=1. Specifically, *η* is the log‐odds ratio for *p*_*t*_ relative to *p*_*c*_. For fixed (*r*_*c*_,*r*_*t*_) and suppressing dependence on the data, write the likelihood *L*(*ζ*,*η*;*r*_*c*_,*r*_*t*_) as

$$\begin{array}{ccc} L(\zeta ,\eta ;r_{c},r_{t})= & \underset{\tau =t,c}{\prod }{\left(\frac{\exp(\zeta +\eta z_{\tau })}{1+\exp(\zeta +\eta z_{\tau })}(1-e^{\gamma_{\tau }}\pi_{\tau })\right)}^{y_{\tau }} & \\ & \times {\left(\frac{1}{1+\exp(\zeta +\eta z_{\tau })}(1-\pi_{\tau })\right)}^{m_{\tau }-y_{\tau }}\\ & \times {\left(\frac{\exp(\zeta +\eta z_{\tau })}{1+\exp(\zeta +\eta z_{\tau })}e^{\gamma_{\tau }}\pi_{\tau }+\frac{1}{1+\exp(\zeta +\eta z_{\tau })}\pi_{\tau }\right)}^{n_{\tau }-m_{\tau }}\\ = & \underset{\tau =t,c}{\prod }e^{(\zeta +\eta z_{\tau })y_{\tau }}{\left(1+e^{\zeta +\eta z_{\tau }}\right)}^{-n_{\tau }}{\left(1+e^{\zeta +\eta z_{\tau }+\gamma_{\tau }}\right)}^{n_{\tau }-m_{\tau }}H(\gamma_{\tau },\pi_{\tau }), \end{array}$$

where *H*(·,·) is a function of the data and the (assumed) known parameters *γ*_*τ*_ and *π*_*τ*_. The log‐likelihood is

$$\log L(\zeta ,\eta ;r_{c},r_{t})=\underset{\tau =t,c}{\sum }(\zeta +\eta z_{\tau })y_{\tau }-n_{\tau }\log\left(1+e^{\zeta +\eta z_{\tau }}\right)+(n_{\tau }-m_{\tau })\log\left(1+e^{\zeta +\eta z_{\tau }+\gamma_{\tau }}\right)+\log H(\gamma_{\tau },\pi_{\tau }).$$

For specified values of (*r*_*c*_,*r*_*t*_), we can write the derivatives of the log‐likelihood with respect to *ζ* and *η* as

(4) $$\begin{array}{ccc} U_{\zeta }(\zeta ,\eta ;r_{c},r_{t})= & \frac{\partial \log L(\zeta ,\eta ;r_{c},r_{t})}{\partial \zeta } & \\ = & \underset{\tau =t,c}{\sum }y_{\tau }-n_{\tau }\frac{e^{\zeta +\eta z_{\tau }}}{1+e^{\zeta +\eta z_{\tau }}}+(n_{\tau }-m_{\tau })\frac{e^{\zeta +\eta z_{\tau }+\gamma_{\tau }}}{1+e^{\zeta +\eta z_{\tau }+\gamma_{\tau }}}\\ = & \underset{\tau =t,c}{\sum }y_{\tau }-n_{\tau }p_{\tau }+(n_{\tau }-m_{\tau })\frac{r_{\tau }p_{\tau }}{1-p_{\tau }+r_{\tau }p_{\tau }} \end{array}$$

and

(5) $$\begin{array}{ccc} U_{\eta }(\zeta ,\eta ;r_{c},r_{t})= & \frac{\partial \log L(\zeta ,\eta ;r_{c},r_{t})}{\partial \eta } & \\ = & y_{t}-n_{t}\frac{e^{\zeta +\eta }}{1+e^{\zeta +\eta }}+(n_{\tau }-m_{\tau })\frac{e^{\zeta +\eta +\gamma_{t}}}{1+e^{\zeta +\eta +\gamma_{t}}}\\ = & y_{t}-n_{t}p_{t}+(n_{\tau }-m_{\tau })\frac{r_{t}p_{t}}{1-p_{t}+r_{t}p_{t}}. \end{array}$$

To conduct the score test of *H*_0_, we set *p*_*t*_=*p*_*c*_=*p* in (4), and solve for $\hat{p}(r_{c},r_{t})$, or equivalently $\hat{\zeta }(r_{c},r_{t})$. The test statistic for *H*_0_ is $U_{\eta }(\hat{\zeta },0;r_{c},r_{t})$. Letting

$$V_{\tau }(r)=n_{\tau }p(1-p)-(n_{\tau }-m_{\tau })\frac{rp(1-p)}{{\left(1-p+rp\right)}^{2}},$$

then the variance of $U_{\eta }(\hat{\zeta }(r_{c},r_{t}),0;r_{c},r_{t})$ can be estimated by $V(r_{c},r_{t})={\left(V_{c}{\left(r_{c}\right)}^{-1}+V_{t}{\left(r_{t}\right)}^{-1}\right)}^{-1}$ where we estimate *p* by $\hat{p}(r_{c},r_{t})$. For fixed (*r*_*c*_,*r*_*t*_), the *Z*‐statistic for testing *H*_0_ is

(6) $$Z(r_{c},r_{t})=-\frac{U(\hat{\zeta }(r_{c},r_{t}),0;r_{c},r_{t})}{\sqrt{V(r_{c},r_{t})}},$$

where the sign is chosen so that *Z*(*r*_*c*_,*r*_*t*_)>0 corresponds to lower mortality in group *t*. Note that when *r*_*c*_=*r*_*t*_=1, this is exactly the complete case *Z*‐statistic, $\tilde{Z}$ given by (3).

For a given plausibility region, R, we will reject *H*_0_ if

$$\underset{(r_{c},r_{t})\in R}{\inf}Z(r_{c},r_{t})\geq 1.96.$$

In other words, we reject *H*_0_ if *Z*(*r*_*c*_,*r*_*t*_) ≥ 1.96 for all (*r*_*c*_,*r*_*t*_) in R.

For illustration, suppose we have the hypothetical data shown in Table 2.

**Table 2:** Example two‐by‐two table with missing responses

	Dead	Alive	Missing	Total
*c*	38	51	11	100
*t*	21	70	9	100

We make the following observations.

1. Overall, responses are missing for 9.5% of subjects.
2. Using the complete data, $\tilde{Z}=2.80$, *p*=0.005 (two sided), with lower failure rate among complete cases in group *t*.
3. Under the “best case,” in which we assume that all group *t* subjects are successes and all group *c* subjects fail, $\tilde{Z}=4.15$, *p*=0.00003.
4. Under the “worst case,” in which we assume that all group *t* subjects fail and all group *c* subjects are successes, $\tilde{Z}=1.19$, *p*=0.23.
5. If the worst case is *plausible*, then we are unable to conclude that the treatment *t* is beneficial relative to treatment *c*.

**Figure 3:**
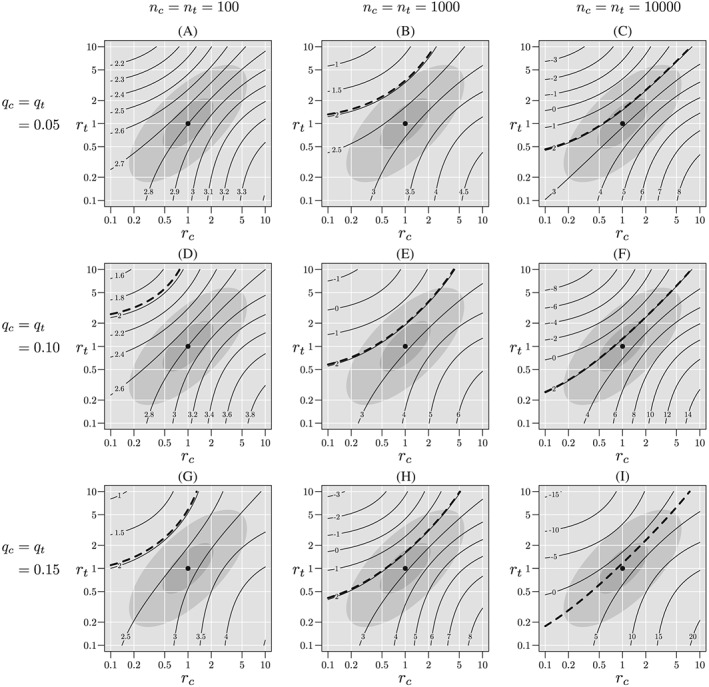
Sensitivity analyses for tables with approximately the same complete‐case *Z* statistic, but differing sample sizes and missingness probabilities. The light gray ellipse corresponds to the skeptical plausibility region, $R_{5}$, and the darker gray ellipse corresponds to the optimistic region, $R_{2}$. The heavy dashed contour line corresponds to *Z*=1.96. If this line is contained in the plausible region, we cannot reject *H*_0_ at the (one‐sided) 0.025 level

A sensitivity analysis for this table is shown in Figure 3, panel D. Within the “optimistic” region, $R_{2}$, the minimum *Z*(*r*_*c*_,*r*_*t*_) is about 2.6, whereas within the “skeptical” region, $R_{5}$, the minimum *Z*(*r*_*c*_,*r*_*t*_) is about 2.3. In either case, *H*_0_ is rejected and the result can be considered robust.

## 6 IMPACT OF SAMPLE SIZE ON SENSITIVITY ANALYSIS

In this section, we consider the impact of sample size on inference using sensitivity analysis. Table 3 shows a series of two‐by‐two tables with a common (approximately) complete case *Z*‐statistic, $\tilde{Z}=2.80$ (two‐sided *p*=0.005). If we use the *Z*‐statistic to assess the strength of evidence against the null hypothesis, then, based solely on the complete case analysis, each of these tables provides essentially the same amount evidence against the null. On the other hand, if one measures distance from the null by, say the odds ratio, the complete‐case odds ratio in these tables becomes closer to one as the sample size increases. Nonetheless, when *H*_0_ is true, all these tables represent the same quantile of the distribution of $\tilde{Z}$ when the null hypothesis is true.

**Table 3:** Hypothetical tables with common complete‐case *Z*‐statistic of $\tilde{Z}=2.80$ and varying sample sizes and missingness proportions. The columns labeled “ $R_{2}$?” and “ $R_{5}$?” indicate whether *H*_0_ is rejected for the optimistic and skeptical regions respectively

							Complete case		
Table	*n*_*c*_=*n*_*t*_	% Missing	Dead *c*	Missing *c*	Dead *t*	Missing *t*	Odds ratio	$R_{2}$?	$R_{5}$?
a	100	5.5	35	6	18	5	0.39	Y	Y
b	1000	5.3	302	54	247	53	0.75	Y	Y
c	10 000	5.2	3007	495	2812	551	0.92	N	N
d	100	10.0	38	11	21	9	0.40	Y	Y
e	1000	10.4	301	105	247	103	0.75	Y	N
f	10 000	10.2	2787	1017	2611	1030	0.91	N	N
g	100	15.0	34	18	19	12	0.39	Y	Y
h	1000	15.0	258	155	209	146	0.74	N	N
i	10 000	15.0	2592	1518	2438	1475	0.91	N	N

The missingness rates range from approximately 5% to 15% and the sample sizes are 100, 1000, and 10 000 per group. The first column links each table to a panel in Figure 3.

Figure 3 shows contours of *Z*(*r*_*c*_,*r*_*t*_) for each of the tables in Table 3. Tables for which the heavy dashed line, corresponding to *Z*(*r*_*c*_,*r*_*t*_)=1.96, intersects a plausible region are those for which *H*_0_ cannot be rejected at the one‐sided 0.025 level. For each of the tables for *n*_*c*_=*n*_*t*_=100, the critical line remains outside both plausible regions, so the result can be considered robust. For *n*_*c*_=*n*_*t*_=1000, the result is robust for approximately 5% missingness and 10% missingness using the optimistic region, $R_{2}$, but is otherwise not robust. For *n*_*c*_=*n*_*t*_=10 000, the result is not robust for any degree of missingness using either plausible region.

## 7 ADJUSTMENTS TO SAMPLE SIZE

In this section, we assume that a plausible region is chosen before study start, and that the sensitivity analysis given in Section 5 will be used at trial completion. As demonstrated in the previous section, a larger difference between groups in complete case failure rates is required when a robust sensitivity analysis is used than would be required with complete data. Hence, power will be reduced unless the sample size is increased to account for expected missingness rates. Let *n* ^∗^ denote the sample size per group with 1:1 randomization required to achieve power 1−*β* assuming that no data will be missing. Given some *plausible region*, R, suppose that $(R_{c},R_{t})\in R$ and let ${\mathrm{Pr}}_{\left(R_{c},R_{t}\right)}$ denote probability under the induced distribution for Table 1 assuming that (*R*_*c*_,*R*_*t*_) is true for fixed (*π*_*c*_,*π*_*t*_).

To preserve the desired power, 1−*β*, for the given R, the per‐group sample size *n*=*n*_*c*_=*n*_*t*_ should be chosen so that

(7) $$\underset{(R_{c},R_{t})\in R}{\max}{\text{Pr}}_{\left(R_{c},R_{t}\right)}\left\{\underset{(r_{c},r_{t})\in R}{\min}Z(r_{c},r_{t})<Z_{1-\alpha /2}\right\}=\beta .$$

We also define the *inflation factor* to be the ratio *n*/*n* ^∗^.

Note that under MAR, R={(1,1)}, and if *q*_*t*_=*q*_*c*_=*q*, Equation (7) yields the “usual” inflation factor of 1/(1−*q*).^11^ That is, if we expect a fraction *q* to be missing, the number subjects expected to be not missing is *n*(1−*q*). Setting this equal to *n* ^∗^ gives the “usual” result. When MAR is not satisfied, so R is larger than the singleton {(1,1)}, the inflation factor will be larger than 1/(1−*q*). Furthermore, if R is large enough, it is possible that no solution to (7) exists, and the desired power is unattainable. In this case, there exists (*r*_*c*_,*r*_*t*_) and (*R*_*c*_,*R*_*t*_) such that ${\mathrm{Pr}}_{\left(R_{c},R_{t}\right)}\{Z(r_{c},r_{t})<Z_{1-\alpha /2}\}\geq 1-\alpha /2$ for all *n*>0.

Note further that the pair (*r*_*c*_,*r*_*t*_) that yields the minimum in Equation (7) is a random variable and computing the exact distribution of the $\min_{\left(r_{c},r_{t}\right)}Z(r_{c},r_{t})$ is analytically intractable, most easily done by computationally intensive simulation. Thus, we will use the following approximation that can be shown by simulation to be quite accurate. By exchanging the order of expectation and minimization in (7), define $\theta_{R}(r_{c},r_{t})$ by

$$\theta_{R}(r_{c},r_{t})=\underset{(R_{c},R_{t})\in R}{\min}E_{\left(R_{c},R_{t}\right)}Z(r_{c},r_{t}),$$

where $E_{\left(R_{c},R_{t}\right)}$ is expectation when (*R*_*c*_,*R*_*t*_) is true. For sample size *n* ^∗^ and a specified alternative hypothesis and missingness probabilities, we have that the inflation factor is approximately

$$\frac{n}{n^{*}}={\left[\frac{Z_{1-\alpha /2}+Z_{1-\beta }}{\min_{(r_{c},r_{t})\in R}\theta_{R}(r_{c},r_{t})}\right]}^{2}.$$

**Figure 4:**
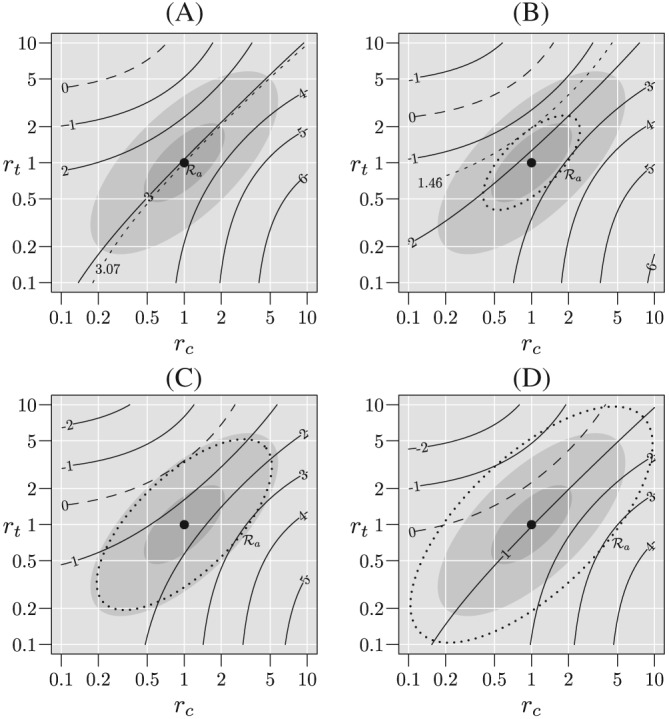
Contours of $\theta_{R}$ given that the true (*r*_*c*_,*r*_*t*_) lies within the plausible region $R_{a}$ for *n* ^∗^=1000, *p*_*c*_=0.3, *p*_*t*_=0.236, and *q*_*c*_=*q*_*t*_=0.10. In panel A missing at random holds, so that the $R_{a}$ is the singleton, {(1,1)} and for panels B, C, and D $R_{a}$ is shown by the dotted ellipse. For panel B, the contour for $Z_{R_{a}}=0$ is excluded from $R_{a}$ and there exists a sample size for which the desired power is possible. For panel C, the contour for $Z_{R_{a}}=0$ is tangent to the boundary of $R_{a}$ and therefore $R_{a}$ corresponds to the “infinite inflation boundary” and the desired power cannot be achieved. For panel D, the contour for $Z_{R_{a}}=0$ crosses the boundary of $R_{a}$ and the desired power cannot be achieved

Figure 4 shows contours of $\theta_{R}(r_{c},r_{t})$ when *q*_*c*_=*q*_*t*_=0.10, *p*_*c*_=0.3, and *p*_*c*_=0.236, for which *n*_*c*_=*n*_*t*_=*n* ^∗^=1000, and an ascending sequence of $R_{a}$. In panel A, *a*=0 and MAR holds, so R={(1,1)}, and $\theta_{R}(1,1)=3.07$. Note that, in this case, the inflation factor is [3.24/3.07]^2^=1.11=1/(1−.1) so the “usual” adjustment applies. In panel B, *a*=2.30, $\min_{(r_{c},r_{t})\in R}\theta_{R}(r_{c},r_{t})=1.46$, and the inflation factor is (3.24/1.46)^2^=4.92. In panel C, $\min_{(r_{c},r_{t})\in R}\theta_{R}(r_{c},r_{t})=0$, and the solution to (7) does not exist. In this case, the dotted ellipse is the *infinite inflation boundary*. That is, for any R that contains this region, it will be impossible to adequately power a trial to account for the expected degree of missingness and plausible deviations from MAR. In panel D, $\min_{(r_{c},r_{t})\in R}\theta_{R}(r_{c},r_{t})<0$, and there exists $(r_{c},r_{t})\in R$ and $(R_{c},R_{t})\in R$ such that ${\mathrm{Pr}}_{\left(R_{c},R_{t}\right)}\{Z(r_{c},r_{t})<Z_{1-\alpha /2}\}\to 1$ as *n*→*∞*. Again, in this case, adequate power is unattainable.

**Figure 5:**
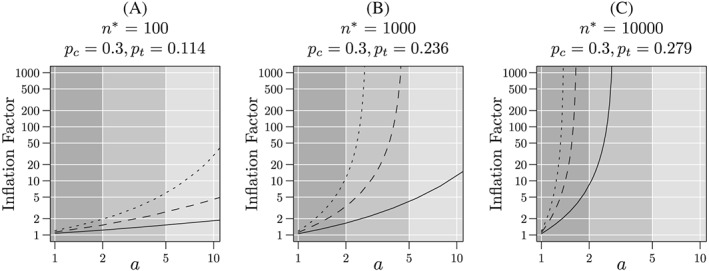
Inflation factor as a function of plausible region, $R_{a}$, for missingness probabilities of 5%, 10% and 15%, and “nominal” sample sizes of 100, 1000, and 10 000. The “nominal” sample size is the number of subject per group required to achieve 90% power for a one‐sided 0.025‐level test assuming no missing responses. The failure probability in the control group is *p*_*c*_=0.3 and the failure probabilities in the experimental group are *p*_*t*_=0.116, 0.236, and 0.279 for 100, 1000, and 10000 respectively. Solid lines correspond to *q*=0.05, dashed lines to *q*=0.10, and dotted lines to *q*=0.15. The dark and medium gray regions correspond to the “skeptical” and “optimistic” regions, respectively

Figure 5 shows the inflation factor as a function R for various values of *n* ^∗^ and missingness fractions when *p*_*c*_=0.3. In this figure, we consider regions $R_{a}$ for 1 ≤ *a* ≤ 10. The “skeptical” and “optimistic” regions are given by *a* ≤ 5 and *a* ≤ 2 respectively and shown by the shaded regions in this figure. Note that the inflation factor for *a*=1 corresponds to the “usual” adjustment. In panel A, for $R_{2}$, the “optimistic” region, the inflation factor is approximately 1.2 when *q*=0.05, 1.5 when *q*=0.10 and 2.0 when *q*=0.15. That is, with 15% missingness, a doubling of the sample size is required, whereas the “usual” inflation factor is 1/0.85=1.18. For $R_{5}$, the “skeptical” region, inflation factors are approximately 1.5, 2.7, and 6.1 for *q*=0.05, 0.10, and 0.15, respectively. In panel B, for $R_{2}$, inflation factors are approximately 1.7, 3.4, and 12.0 for *q*=0.05, 0.10, and 0.15 respectively. For $R_{5}$, it is approximately 4.2 when *q*=0.05, but unattainable when *q* ≥ 0.10. In panel C, the inflation factor is approximately 8.7 for $R_{2}$ and *q*=0.05 but unattainable when *q* ≥ 0.10 and for $R_{5}$.

## 8 DISCUSSION

In this article, we discuss the impact of missing responses on the causal conclusions that can be drawn from a randomized controlled trial. Unless we can be confident that responses are missing at random, a complete case analysis does not have a valid interpretation as an assessment of the causal effect of treatment on the underlying risk of interest. We have shown that causal inference from the complete case analysis breaks down not only as missingness rates increase but, because with larger sample sizes, we can detect smaller differences between groups, as sample sizes increase such analyses are *less* robust to even small amounts of missingness.

Because the missing data mechanism will always be uncertain, sensitivity analyses should be used to formally assess robustness to *plausible* missing data mechanisms. Here, we have chosen to consider sensitivity analyses using likelihood‐based inference and fixed plausible regions for the missingness mechanism. Other approaches are possible, but the general behavior should be independent of the approach. For example, one might consider multiple imputation, where the imputations are generated from a range of MNAR models. However, because our likelihood‐based models implicitly impute the missing responses, inference using similar models coupled with explicit imputation should yield virtually identical results. Furthermore, as noted earlier, by weighting pairs, (*r*_*c*_,*r*_*t*_), using a probability distribution over a region R, one could conduct sensitivity analyses with a more Bayesian flavor. One can also, in principle, incorporate baseline covariates into these analysis, but we have not investigated that approach.

We have shown that for fixed levels of statistical significance and fixed plausible regions, trials with larger sample sizes are less robust than trials with small sample sizes. In small trials, large observed differences are required to achieve high levels of statistical significance and these large differences are less likely to be a result of missingness. For large trials, high levels of statistical significance can be attained with relatively small observed differences; however, these small differences are more easily contaminated by data that are missing not at random.

Finally, when it is expected from the design stage that sensitivity analyses will be used with a specified plausible region and expected missingness rates, sample size increases relative to those required in the absence of missingness are required to maintain adequate power. Furthermore, we have shown that, unless expected missingness rates are relatively low or plausible regions relatively small, the required sample size adjustments may be quite dramatic or, in the extreme, achieving the desired power may be impossible regardless of sample size.

We have several additional comments. First, we have considered the simplest possible case of binary responses. For responses with more than two levels, missingness models rapidly grow quite complex and regions of plausible deviations from MAR quickly become high dimensional. In these cases, parsimonious families are required to capture enough of the space of deviations to provide robust results. It is likely that, for other data types, the findings shown here will only become *more* extreme, but additional work will be required to demonstrate this.

Second, the choice of plausible region is both subjective and difficult. The fundamental difficulty is, of course, that the data required so assess the missing data mechanism is, by definition, missing not only in the particular trial that we may be conducting but also in all historical trials. In this paper, for the purpose of illustration, we have arbitrarily chosen example plausible regions. Nordheim,^12^ using a similar approach albeit in a different setting, states “It is anticipated that in almost all cases researchers will know if [*r*_*τ*_]<1 or [*r*_*τ*_]>1 and in most cases should be able to restrict [*r*_*τ*_] to a narrower interval.” Whether this is generally true in RCTs is unclear and we offer no guidance in this article.

Consistent with previous recommendations, we recommend that

1. missingness rates should be kept as low as possible, even 10%, may be too high, especially in *large* trials;
2. robust sensitivity analyses are essential for assessing the potential impact of missingness;
3. if robust sensitivity analyses are to be performed and trials required to stand up to scrutiny, much larger sample sizes will be required. The usual adjustments are likely far to small;
4. deliberately introducing missingness through “per‐protocol” or “adherers‐only” analyses not only does not properly address the concerns raised by incomplete adherence but may easily render analyses completely uninterpretable.

With these recommendations in mind, we note that clinical trials in humans are not conducted primarily for the benefit of the investigators or study sponsors, but rather for the benefit of the broader scientific community and, most important, the target population of potential recipients of the interventions under study. As such, design and analysis decisions should be made so that the trial results are convincing, not just to sponsors or investigators, but to an informed, reasonable skeptic from this community—someone who, without convincing evidence, will not accept the conclusion. For example, sponsors should invest adequate resources into minimizing the extent of missing data and not simply accept it as inevitable. Modest improvements in missingness rates may dramatically improve the credibility of findings and the return on investment might be the difference between a failed inconclusive trial and a convincing result.

Conversely, the broader community needs to establish criteria by which results can be considered robust, providing sponsors and investigators a starting point. However, as noted, these criteria cannot be based on prior data because, by definition, the required data does not, and never will, exist.

In our opinion, our findings are sobering. While the problem of missing data has historically been a major concern, the consequences may be more severe than previously understood. Missingness rates well in excess of the maximum of 15% that we have considered in this article are commonplace. It is likely that, were robust sensitivity analyses were performed, the findings of a great many trials would be called into question. Clearly, more research into the treatment, and maybe more important, the prevention of missing data is required.

## Supporting information

SIM_8428‐Supp‐00001‐example.R

## References

[1] Wittes JT . Missing inaction: preventing missing outcome data in randomized clinical trials. J Biopharm Stat. 2009;19:957‐68. 20183458 10.1080/10543400903239825

[2] Fleming TR . Addressing missing data in clinical trials. Ann Intern Med. 2011;154:113‐117. 21242367 10.1059/0003-4819-154-2-201101180-00010 PMC3319761

[3] National Research Council (US) Panel on Handling Missing Data in Clinical Trials . The Prevention and Treatment of Missing Data in Clinical Trials. Washington, DC: The National Academies Press; 2010. 24983040

[4] Little RJ , D'Agostino R , Cohen ML , et al. The prevention and treatment of missing data in clinical trials. N Engl J Med. 2012;367(14):1355‐1360. 23034025 10.1056/NEJMsr1203730 PMC3771340

[5] O'Neill RT , Temple RJ . The prevention and treatment of missing data in clinical trials: an FDA perspective on the importance of dealing with it. Clin Pharmacol Ther. 2012;91(3):550‐554. 10.1038/clpt.2011.340 22318615

[6] Rubin DB . Estimating causal effects of treatment in randomized and nonrandomized studies. J Educ Psychol. 1974;66:688.

[7] Holland PW . Statistics and causal inference. J Am Stat Assoc. 1986;81:945‐960.

[8] Rubin DB . Inference and missing data. Biometrika. 1976;63:581‐590.

[9] Little RJA , Rubin DB . Statistical Analysis With Missing Data. Hoboken, NJ: John Wiley & Sons; 2002.

[10] Rubin DB . Bayesian inference for causal effects: the role of randomization. Ann Stat. 1978;6(1):34‐58.

[11] Donner A . Approaches to sample size estimation in the design of clinical trials—a review. Statist Med. 1984;3:199‐214. 10.1002/sim.4780030302 6385187

[12] Nordheim EV . Inference from nonrandomly missing categorical data: an example from a genetic study on Turner's syndrome. J Am Stat Assoc. 1984;79(388):772‐780.

